# Low-Cost Automated Design of Compact Branch-Line Couplers

**DOI:** 10.3390/s20123562

**Published:** 2020-06-23

**Authors:** Adrian Bekasiewicz

**Affiliations:** Faculty of Electronics, Telecommunications and Informatics, Gdansk University of Technology, 80-233 Gdansk, Poland; bekasiewicz@ru.is; Tel.: +48-583-472-019

**Keywords:** automated design, simulation-driven design, circuit miniaturization, branch-line couplers, surrogate-based optimization, Internet of Things

## Abstract

Branch-line couplers (BLCs) are important components of wireless communication systems. Conventional BLCs are often characterized by large footprints which make miniaturization an important pre-requisite for their application in modern devices. State-of-the-art approaches to design compact BLCs are largely based on the use of high-permittivity substrates and multi-layer topologies. Alternative methods involve replacement of transmission-line sections of the circuit, with their composite counterparts, referred to as compact cells (CCs). Due to the efficient use of available space, CC-based couplers are often characterized by small footprints. The design of compact BLCs is normally conducted based on engineering experience. The process is laborious and requires many adjustments of topology followed by manual or, semi-automatic tuning of design parameters. In this work, a framework for low-cost automated design of compact BLCs using pre-defined CCs is proposed. The low cost of the presented design technique is ensured using equivalent-circuit models, space mapping correction methods, and trust-region-based local optimization algorithms. The performance of the framework is demonstrated based on three examples, concerning the design of unequal-power split coupler, comparison of automatically generated compact BLCs, as well as rapid re-design of the coupler for different substrates. Furthermore, the approach has been benchmarked against the state-of-the-art methods for low-cost design of circuits.

## 1. Introduction

Branch-line couplers (BLCs) belong to the key components of many microwave devices including beamforming networks [1,2], balanced mixers [3,4], amplifiers [5,6], multiplexers [7,8], and others. Conventional BLCs are characterized by large dimensions as they consist of two orthogonal pairs of 90° transmission lines (TLs). Miniaturization is an important pre-requisite to facilitate the application of couplers in modern communication systems, such as portable [9,10], or wearable electronics [11,12], but also Internet of Things (IoT) devices [13,14].

Conventional approaches to couplers miniaturization include utilization of high-permittivity substrates [15], or replacement of TLs with their lumped-element equivalents [16]. An interesting alternative is in the replacement of conventional TLs by so-called compact cells (CCs). The latter are normally implemented using high-impedance lines [17], or a combination of high- and low-impedance sections [16], but also as appropriately folded TLs [18], coupled sections featuring unequal length [19], or fractal-based topologies [20]. Due to the reduced phase velocity (also referred to as slow-wave effect) [21,22,23,24], CCs are shorter than conventional transmission lines which is advantageous for design of miniaturized couplers [24]. The state-of-the-art CC-based BLCs offer size-reduction rates ranging from nearly 40% to over 85% [24,25,26,27,28,29]. On the other hand, a small size is often achieved at the expense of degraded electrical properties (most notably in terms of narrower bandwidth or deviation of power split from the desired value) [26,30]. However, couplers featuring small size and high performance have also been reported in the literature [16,31,32]. Furthermore, owing to low-pass properties of CCs, compact BLCs are often capable of suppressing harmonic frequencies [15,24,28,30,31].

Strategies for design of cell-based miniaturized BLCs fall into two categories: (i) development of CCs tailored for specific problem and (ii) selection of the cells from the pre-defined library. The first approach benefits from high miniaturization rates [24,29,33], but it is also laborious and time-consuming [24,29]. The process follows the concept of cognitive design [34]. It involves a manual experience-driven development of cells, but also maintaining their geometrical consistency with adjacent components. The latter is often achieved through definition of complex geometrical relations between individual cells [35,36]. Reuse of pre-defined CCs simplifies BLC development [16,32,37], but at the expense of more modest miniaturization rates. It is worth noting that, for the majority compact of BLCs, miniaturization is achieved as a by-product of performance-oriented tuning rather than dedicated design approach [31,33,38]. Nonetheless, small couplers, obtained as a result of explicit optimization w.r.t. electrical performance and size, have also been reported [39,40].

The selection of cells that ensure high coupler performance while maintaining its small size is a difficult task. The main challenge involves developing a method for unequivocal comparison of CCs [34]. An attempt to address the problem was proposed in [16], where cells are compared in terms of the slow-wave factor [41]. A more generic method concerning pre-optimization of cells with respect to specifications followed by their miniaturization was considered in [24]. Both approaches disregard the importance of optimization for controlling size and performance of the CC-based coupler. Moreover, the computational cost of the methods is high as they exploit only electromagnetic (EM) simulations for evaluation of designs. As indicated in [32,37], this is undesired because performance of the cell can be approximated via simulations of its equivalent-circuit model. 

Performance-related tuning of compact BLCs is often a subject to strict requirements concerning, among others, reflection, isolation, phase shift, or bandwidth. Conventional approaches for tuning of structure performance—based on parametric studies followed by visual inspection of responses—are unsuitable for controlling multiple design criteria at a time [30,33]. From this perspective, the application of numerical optimization is a mandatory step of BLC design [42,43,44,45]. Unfortunately, conventional algorithms require a large number of model evaluations to find the optimum solution. At the same time, due to geometrical complexity (particularly in terms of tightly packed cells and multiple cross-couplings between individual components), accurate evaluation of compact BLCs is possible only by means of high-fidelity EM simulations [16,38]. Consequently, the application of conventional optimization routines for the design of miniaturized couplers is a numerically demanding task [35].

Challenges related to high cost of compact BLC design can be addressed using surrogate-based optimization (SBO) [46,47,48,49,50,51,52]. In SBO, the computational burden of numerical optimization is shifted to a surrogate model. The latter consists of a numerically cheap, less accurate low-fidelity model and an appropriate correction layer, which is determined based on occasionally acquired high-fidelity EM simulation data [48,52]. SBO approaches that are suitable for the design of compact microwave circuits exploit both physics-based and functional approximation surrogates [32,47,48]. They include variants of space mapping (SM) [48,53,54], but also feature-based optimization techniques [55,56]. Surrogate-assisted methods tailored to design of miniaturized structures were also considered in the literature [36,37,38,53,54]. A sequential SM technique, where iterative replacement of conventional TL sections with their CC-based equivalents, is followed by surrogate-based refinement of structure response was proposed in [53]. A more advanced method—where correction of the low-fidelity model was first performed at the level of individual cells, and then at the level of the assembled circuit—was introduced in [54]. Another approach, reported in [32], allows for screening of cells based on their performance w.r.t. reference TLs. Then, the selected CCs undergo further optimization using space-mapping-corrected local approximation models [32]. In [55], a feature-based EM-driven optimization of compact couplers was considered. The core of the method is the translation of original frequency characteristics into a set of carefully selected feature points representing structure behavior. Less-nonlinear dependence of response features on changes of input parameters—as compared to frequency characteristics—improves convergence of the optimization and reduces its cost [55,57]. Surrogate-assisted design can be also realized using variable-fidelity EM simulation models. Despite higher-cost than for circuit-model-based methods [32,53,54], variable-fidelity approaches proved to be useful for generation of compact cells [38].

The above considerations indicate that a framework for low-cost design of compact couplers should embed the mechanisms for: (i) screening the pre-defined library in order to select suitable CCs (geometry- and performance-wise), (ii) ensuring geometrical consistency of assembled structure, and (iii) optimizing its performance/size-related properties for a range of specifications. From the design automation standpoint the main challenges are related to determination of suitable starting point for cell-level optimization, but also development of techniques that enable low-cost unequivocal comparison of CCs [32,38,40]. Standardized approaches for ensuring geometrical consistency of compact couplers, although available, support only structures that combine CCs and meandered TLs [32,37,38]. For other components, manual definition of constraints and mutual relations between individual cells is required [24,35,54]. Finally, state-of-the-art techniques for low-cost design of couplers are either difficult to setup and unsuitable for automation [32,53], or too expensive to justify their use as a tool for, e.g., the optimization-driven comparison of topologies [35,38]. The implication is that existing methods are dedicated for solving specific narrow classes of problems pertinent to design of compact BLCs. In this regard, a more holistic approach oriented for reliability and simplification of the design process is required.

In this work, a framework for unsupervised design of miniaturized branch-line couplers is presented. The method follows the concept of bottom-up design, where the BLC development is realized in the following steps: (i) selection/optimization of compact cells from pre-defined library, (ii) BLC assembly followed by its two-stage optimization, and (iii) surrogate-assisted fine tuning of the coupler. The proposed method exploits circuit and EM simulation models, low-fidelity model correction schemes, and feature-based representation of the structure responses. The response features are used at the cell- and BLC-model levels. In each design stage, the optimization is performed using a gradient algorithm embedded in a trust-region framework. The introduced methodology is validated using three case studies concerning design of miniaturized coupler with unequal power split, comparison of BLC topologies in terms of achievable miniaturization rates, and rapid re-design of coupler for different substrate materials. The tool has been benchmarked against the other numerically efficient design methods in terms of computational cost and quality of obtained solutions. The average cost of BLC development using the presented methodology amounts to only 17.5 min. Moreover, it is capable of generating topologies featuring miniaturization rates of almost 80 percent.

The remainder of the paper is organized as follows. Section 2 discusses the models and algorithms used in the work. Implementation of the developed framework is explained in Section 3, whereas Section 4 contains numerical verification of the proposed methodology and discussion of the results. Section 5 concludes the paper and indicates the directions for future research.

## 2. Simulation Models and Design Tools

The section describes physics-based simulation models employed in the proposed framework. In order to make the paper self-consistent, a brief explanation of the core methods and algorithms used in the work is also included here. The details concerning implementation of the proposed framework for automated design of couplers are provided in Section 3.

### 2.1. Transmission Line-Based Sections vs. Compact Cells

**Figure 1:**
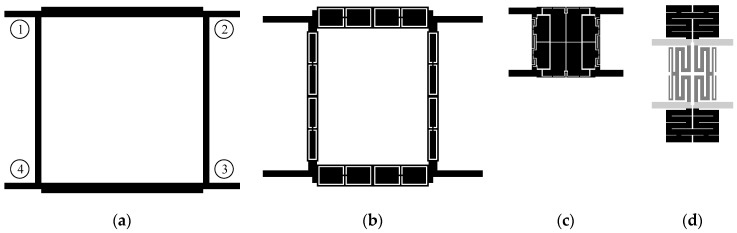
Comparison of microstrip equal-split branch-line couplers in terms of size: (**a**) A conventional structure [58], as well as circuits (**b**) miniaturized using standard symmetrical cells (size reduction: 30%) [53], (**c**) manually developed cells (reduction: 84%) [24], and (**d**) a combination of horizontal (black) and vertical (dark gray) CCs (reduction: 75%). Due to the implementation of couplers on different substrates, miniaturization rates are calculated based on dimensions expressed in terms of guided wavelength [61]. Numbers in circles next to the conventional design denote the enumeration scheme of BLC ports that is used in the work.

Microstrip transmission lines belong to basic building blocks of many conventional microwave circuits, including BLCs. Due to inefficient use of available space, TL-based circuits are often characterized by unacceptably large footprints [24,25]. The problem can be addressed by replacing TLs with compact cells. CCs mimic electrical behavior of transmission lines while offering reduced physical length [16,31]. The cells also offer increased number of degrees of freedom compared to TLs (controlled only through adjustment of electrical length and characteristic impedance [58,59]), which provides additional flexibility in the course of circuit design process [29,38]. As already mentioned, compact BLC can be constructed using off-the-shelf cells (easy to reuse but offer moderate miniaturization rates), or structures developed manually based on the principles of cognitive design (laborious and time consuming but desirable from miniaturization standpoint) [34]. In this work, a balance between simplicity of the design process and achievable size reduction rates is maintained using asymmetrical CCs in appropriate configuration. Specifically, the BLCs considered here combine cells in vertical and horizontal orientations. Such a complementary setup is convenient, as it maintains the geometric consistency of coupler topology using a single parameter that controls separation between the cells. Another benefit is that the cells can be easily interchanged which facilitates rapid generation of various geometries. A conceptual comparison of conventional and miniaturized BLC structures is shown in Figure 1.

### 2.2. Compact Cell and BLC Models

**Figure 2:**
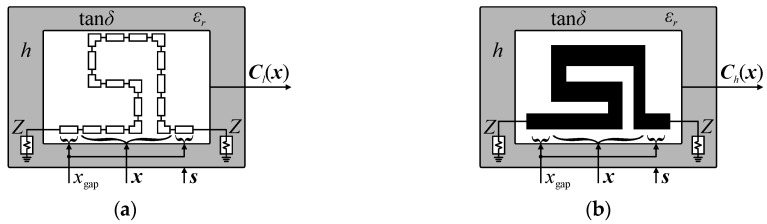
Conceptual illustration of a compact cell (here, in horizontal configuration): (**a**) low-fidelity model ***C****_l_*(***x***) and (**b**) high-fidelity EM-based model ***C****_h_*(***x***). Response of the cell is controlled using input variables vector ***x***, whereas parameters *x*_gap_ and ***s*** = [*Z_H_ h ε_r_ t* tan*δ*]*^T^* maintain separation between adjacent cells and control simulation conditions.

Let ***C****_f.o_*(***x****_o_*,*x*_gap_,***s****_o_*) be the response (*S*-parameters versus frequency) of a generic model of the compact cell. Here, ***x****_o_* represents the vector of model input parameters, whereas *x*_gap_ is a global, fixed parameter used to maintain desired separation between adjacent CCs that constitute the assembled BLC structure. The vector ***s****_o_* = [*Z_o_ h ε_r_ t* tan*δ*]*^T^* represents setup parameters, such as impedance and substrate properties (i.e., height, permittivity, metallization thickness, and loss tangent). The subscript parameters *f* ∈ {*l*, *h*, *s*} and *o* ∈ {*H*, *V*} represent fidelity of the model and configuration of the cell, respectively. In other words, replacement of *f* with *l*, *h*, or *s* means that the structure is evaluated using either the low-fidelity equivalent circuit model, the high-fidelity EM model, or surrogate model. Similarly, models and parameter vectors with *H* or *V* in subscript indicate cells in horizontal and vertical configurations (cf. Figure 1), respectively. For simplicity of notation, let ***C****_f_*(***x***) = ***C****_f.o_*(***x***) = ***C****_f.o_*(***x****_o_*,*x*_gap_,***s****_o_*). External dimensions of individual CC are defined as [*a_o.w_ a_o.h_ x*_gap_]*^T^* = *A_C_*(***C****_f_*(***x****_o_*)), where *a_o.w_* and *a_o.h_* stands for width and height of the cell, whereas *A_C_* is the function that calculates the size. Conceptual illustration of the cell in horizontal configuration is shown in Figure 2. 

The BLC response is calculated based on the transmission line theory from complex electrical characteristics of individual cells [58,59,60]. A MATLAB-based implementation of the considered coupler model supports seamless and automated switching between the CCs pre-defined in the library, as well as unsupervised evaluation of the resulting topologies. The coupler responses are calculated sequentially between selected pairs of *ab* ports (see Figure 1 for ports enumeration) as,

(1) $$B_{ab}\left(y\right)=V_{ab}\left(C_{f.H}\left(x_{H},x_{gap},s_{H}\right),C_{f.V}\left(x_{V},x_{gap},s_{V}\right)\right)$$

where ***y*** = [***x****_H_* ***x****_V_*]*^T^*. The function *V_ab_* realizes the electrical connection that is specific for the selected *ab* pair [59]. The response of (1) is of the form ***B***_pair.*ab*_(***y***) = [***B****_aa_*(***y***) ***B****_ab_*(***y***) ***B****_ba_*(***y***) ***B****_bb_*(***y***)]. Note that yielding sufficient information on BLC performance requires evaluation of only *ab* ∈ {12, 13, 14} pairs, i.e., ***B***_pair.12_(***y***) = [***B***_11_(***y***) ***B***_12_(***y***) ***B***_21_(***y***) ***B***_22_(***y***)], ***B***_pair.13_(***y***) = [***B***_11_(***y***) ***B***_13_(***y***) ***B***_31_(***y***) ***B***_33_(***y***)], and ***B***_pair.14_(***y***) = [***B***_11_(***y***) ***B***_14_(***y***) ***B***_41_(***y***) ***B***_44_(***y***)]. Then the selected responses are aggregated into the following matrix:

(2) $$B\left(y\right)=\left[\begin{array}{cccc} B_{11}\left(y\right) & B_{12}\left(y\right) & B_{13}\left(y\right) & B_{14}\left(y\right) \end{array}\right].$$

**Figure 3:**
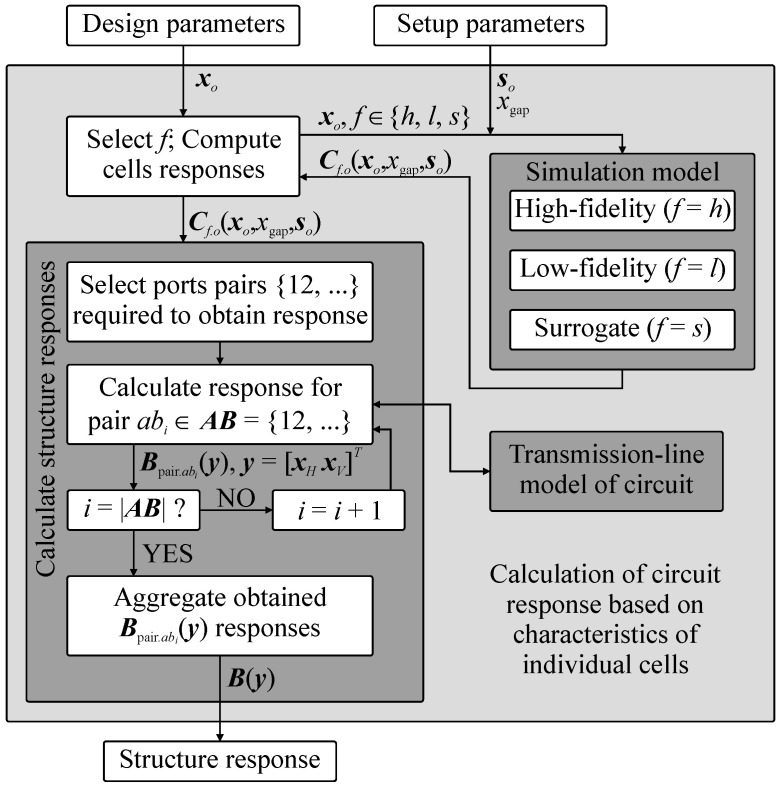
Calculation of structure responses (here, BLC) based on frequency characteristics of its building blocks. Note that all the steps enclosed in a light gray rectangle are controlled by the design framework of Section 3. Hence, the role of the user boils down to the choice of specifications and substrate-related parameters.

It should be stressed out that (1)–(2) support the high-fidelity, low-fidelity and surrogate-based representations of cells, i.e., ***B***(***y***) = ***B****_f_*(***y***), *f* ∈ {*h*, *l*, *s*}. The benefits of the considered model are as follows (i) it operates only on electrical responses and thus supports seamless combination of cells developed using various software packages, (ii) it is universal as the type of the circuit under design (here, BLC) can be changed only through modification of *V_ab_* function, (iii) it can be rapidly re-used to obtain structure responses for various combinations of cells topologies. An important remark is that (2) neglects the cross-coupling effects between the cells which limits its accuracy [35,37]. These, however, are accounted for in a high-fidelity EM model of the assembled coupler (cf. Figure 1), denoted as ***B****_A_*(***y***). The latter is used only at the final stage of the design process. The detailed block diagram that summarizes calculation of BLC responses based on characteristics of individual CCs is shown in Figure 3.

**Figure 4:**
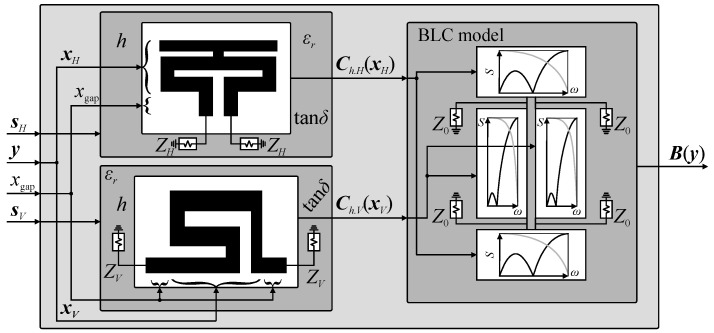
Conceptual illustration of the BLC model (here, ***B***(***y***) = ***B****_h_*(***y***)). The structure response is obtained using transmission-line theory based on characteristics resulting from simulations of individual cells (here, represented in the form of high-fidelity EM models). The model supports cells implemented in the form of EM, equivalent-circuit, or surrogate models. Consistency of coupler geometry is ensured by using cells in horizontal and vertical configurations.

The BLC footprint can be calculated as follows,

(3) $$A_{B}\left(y\right)=\max\left\{a_{H.w},2a_{V.w}+a_{H.g}\right\}\cdot \left(2a_{H.h}+a_{V.h}+2w_{0}\right)$$

where *w*_0_ represents the width of coupler input ports (calculated based on transmission line theory for the given substrate parameters) [58,59]. Conceptual illustration of the considered universal coupler model is shown in Figure 4. 

### 2.3. Problem Formulation

The problem concerning design of the individual cell or the coupler can be formulated as the following non-linear minimization task [62],

(4) $$z^{*}=\arg\underset{z}{\min}U\left(R\left(z\right)\right)$$

where ***R***(***z***) is response of the structure under design obtained for the given vector of design parameters ***z***. Here, *U* represents a scalar objective function. It should be noted that for the cell-level design ***z*** = ***x****_o_* and ***R***(***z***) = ***C****_h.o_*(***x***). Similarly, ***z*** = ***y*** and ***R***(***z***) = ***B***(***y***) when coupler-level design is considered. The goal of (4) is to find the optimal design z* through minimization of the objective function.

### 2.4. Surrogate-Assisted Optimization

Direct solving of (4) is numerically expensive when ***R***(***z***) is evaluated using high-fidelity representation of the structure at hand. Computational cost of the design process can be substantially reduced using surrogate-based optimization. SBO generates a series of approximations, *j* = 1, 2, …, to (4) by solving [62],

(5) $$z^{\left(j+1\right)}=\arg\underset{z}{\min}U\left(R_{s}{}^{\left(j\right)}\left(z\right)\right)$$

Here, ***R****_s_*^(*j*)^(***z***) represents the surrogate model response of the structure under design at *j*th iteration of (5). The surrogate is implemented as the equivalent-circuit model enhanced using the correction layer. The latter is determined based on occasional evaluations of the high-fidelity model which are performed to verify the quality of ***z***^(*j*+1)^ designs [48,62]. The high-fidelity data is also used to gradually improve the ***R****_s_*^(*j*)^(***z***) accuracy by updating the correction layer. In this work, the low-fidelity model correction is applied only at the level of individual cells. The correction layer is implemented as a combination of the implicit space mapping (SM) and frequency scaling [62,63]. The surrogate model of the cell ***R****_s_*^(*j*)^(***z***) = ***C****_s_*^(*j*)^(***x***) is of the following form,

(6) $$C_{s}{}^{\left(j\right)}\left(x\right)=C_{s}{}^{\left(j\right)}\left(x,p^{\left(j\right)}\right)=C_{c}\left(x,x_{c}{}^{\left(j\right)},\alpha^{\left(j\right)}\omega \right)$$

where ***p***^(*j*)^ = [***x****_c_*^(*j*)^ ***α***^(*j*)^]*^T^* is a set of parameters used for adjusting the correction layer. The vector ***x****_c_*^(*j*)^ = [*h*_1_ *h*_2_ … *ε_r1_ ε_r2_ …*]*^T^* represents a set of control variables (also referred to as the preassigned parameters) for implicit space mapping (ISM), i.e., substrate thickness *h* and permittivity *ε_r_*. They can be adjusted to reduce discrepancy between the surrogate and the high-fidelity model responses [63,64]. The component ***α***^(*j*)^***ω*** = *α*_0_^(*j*)^ + *α*_1_^(*j*)^***ω*** represents frequency scaling. It is used to “shift” and/or “stretch/squeeze” response of the surrogate w.r.t. the original frequency sweep ***ω***. 

In each SBO step, concurrent update of the correction layer parameters is performed. The adjustment is realized in the course of a so-called parameter extraction (PE) [48,64]—a curve fitting process oriented towards matching the low-fidelity model response ***C****_l_* to the high-fidelity model ***C****_h_*. Here, PE is defined as the following minimization task:

(7) $$p^{\left(j\right)}=\arg\underset{p}{\min}\left\|C_{h}\left(x^{\left(j\right)}\right)-C_{l}\left(x^{\left(j\right)},p^{\left(j\right)}\right)\right\|.$$

**Figure 5:**
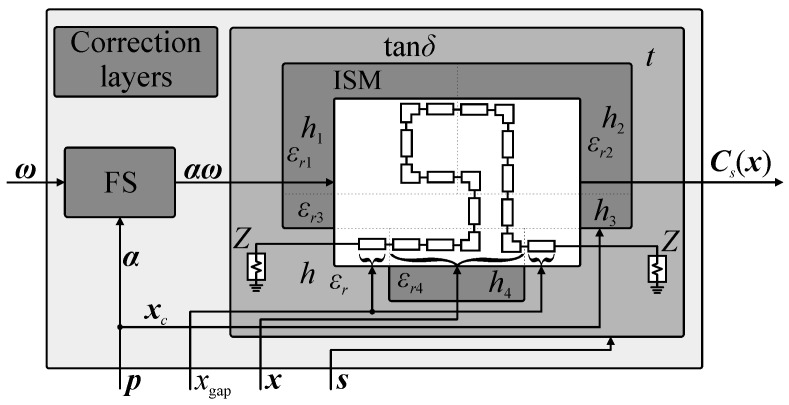
Surrogate model of individual CC—conceptual illustration. The correction layer parameters ***p*** = [***x****_c_* ***α***]*^T^* are adjusted in the course of parameter extraction process. Cell geometry and substrate parameters at the input port are maintained using ***x***, *x*_gap_, and ***s*** parameters.

A conceptual illustration of the CC surrogate with highlight on the low-fidelity model and the correction layer is shown in Figure 5. For more detailed discussion on SBO, as well as low-fidelity model correction methods, see [49,51,54,62,63].

### 2.5. Feature-Based Representation of Structure Responses

One of the main challenges related to development of reliable methods for unsupervised design is that responses of couplers—and the responses of their building blocks—are highly nonlinear functions of frequency [55]. To be considered generic, CCs stored in the library have to support a broad range of substrate and geometry parameters, as well as operate within relatively wide frequency spectrum. Additionally, automated selection of cells should be performed as a result of rigorous optimization process. However, development of objective function that accurately represents cell performance for a wide range of design scenarios and input parameters is difficult. Yet another issue associated with optimization-based cell-selection is the risk that algorithm will get stuck in poor local optimum. The difficulties concerning performance adjustment become even more pronounced when cells are used for construction of structures with complex response characteristics, such as BLCs. 

**Figure 6:**
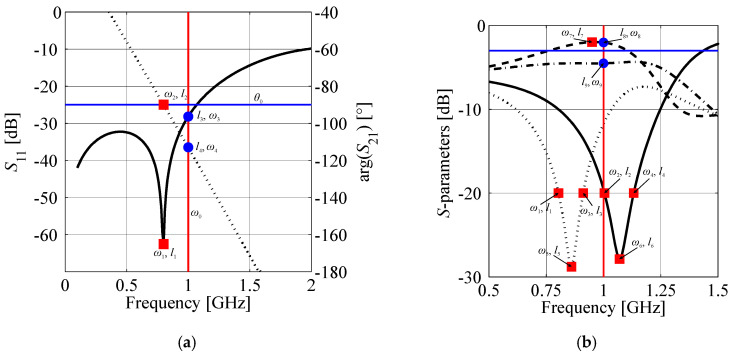
Representation of structure characteristics in the form of feature points: (**a**) individual cell and (**b**) coupler. The squares represent feature points defined w.r.t. level (i.e., frequency point for the given level *l*). The circles are features defined w.r.t. frequency (i.e., response level for the given frequency point *ω*). Parameters *ω*_0_ and *θ*_0_ represent the center frequency and the desired phase shift.

**Figure 7:**
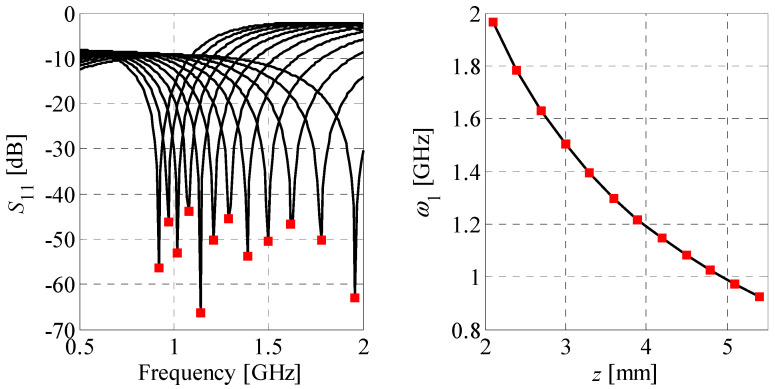
Family of CC characteristics (left-hand side) obtained for an arbitrarily selected line segment *z* in the search space and their corresponding feature-based response (right-hand side) defined w.r.t. level (here, minimum of *S*_11_ in frequency).

**Figure 8:**
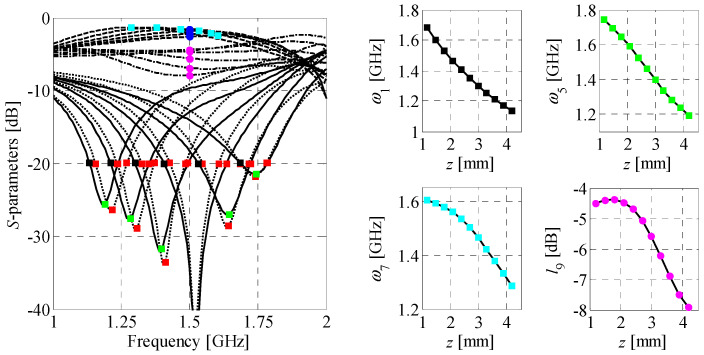
Family of BLC characteristics (left-hand side) obtained for an arbitrarily selected line segment *z* in the search space and their corresponding feature-based responses (right-hand side) defined w.r.t. level (squares) and frequency (circles). Marker colors on frequency plot correspond to marker colors on the selected feature plots.

Mentioned challenges can be addressed by converting frequency responses of the structure into an appropriate domain. Here, this task is realized by reformulating the frequency characteristics to the form of response features [57]. The latter represent each key feature of the structure frequency response using a pair of carefully selected coordinates. Feature points can be defined as follows. Let ***F***(***z***) = *P*(***R***(***z***)) be the response of the model ***R*** expressed in the form of feature points, where *P* is the function that realizes the transformation. The feature-based response of the structure at hand is given by,

(8) $$F\left(z\right)={\left[\begin{array}{c} \omega \left(R\left(z\right)\right)\\ l\left(R\left(z\right)\right) \end{array}\right]}^{T}={\left[\begin{array}{c} \omega \\ l \end{array}\right]}^{T}={\left[\begin{array}{ccc} \omega_{1} & \ldots & \omega_{N}\\ l_{1} & \ldots & l_{N} \end{array}\right]}^{T}$$

where *ω_n_* and *l_n_*, *n* = 1, …, *N* represent the frequency point and its corresponding value of the response (also referred to as level), respectively. As shown in Figure 6, the *n*th coordinate can be defined either for a fixed frequency point, or for a desired level of circuit response. This flexibility makes features a convenient tool for tracking the key changes of structure responses. Another advantage is less non-linear dependency of the features as a function of input parameters compared to original frequency characteristics (cf. Figure 7 and Figure 8). The mentioned properties make this form of data representation an efficient tool for addressing challenges related to unsupervised design of compact couplers. In particular, description of circuit responses in terms of response features improves convergence of the optimization process and reduces its computational cost, as compared to design tasks that involve direct evaluation of frequency responses [56,57]. 

### 2.6. Optimization Engine

The optimization engine used in this work for solving (5) and (7) is a gradient algorithm embedded within a trust-region (TR) framework. It takes the form of [44],

(9) $$z^{\left(j+1\right)}=\arg\underset{z:\left\|z-z^{\left(j\right)}\right\|\leq r^{\left(j\right)}}{\min}U\left(G^{\left(j\right)}(z)\right)$$

where ***G***^(*j*)^(***z***) is the first-order Taylor expansion model constructed at the level of response features,

(10) $$G^{\left(j\right)}\left(z\right)=F_{f}\left(z^{\left(j\right)}\right)+J\left(z^{\left(j\right)}\right)\cdot \left(z-z^{\left(j\right)}\right)$$

Here, ***F****_f_ = P*(***R****_f_*) represents the feature-based responses of the structure at hand obtained at either the low- (*f* = *l*), high-fidelity (*f* = *h*), or surrogate model level (*f* = *s*), respectively. The Jacobian ***J***(***z***^(*j*)^) is obtained using a large-step finite differentiation as [44]

(11) $$J\left(z^{\left(j\right)}\right)={\left[\begin{array}{c} d_{1}{}^{-1}\left(F_{J.f}\left(z^{\left(j\right)}+e_{1}\right)-F_{J.f}\left(z^{\left(j\right)}\right)\right)\\ \vdots \\ d_{D}{}^{-1}\left(F_{J.f}\left(z^{\left(j\right)}+e_{D}\right)-F_{J.f}\left(z^{\left(j\right)}\right)\right) \end{array}\right]}^{T}$$

The responses ***F****_J.f_ = P*(***R****_J.f_*) used for construction of the Jacobian are obtained from the low-fidelity ***R****_J.f_* = ***R****_l_* or surrogate ***R****_J.f_* = ***R****_s_* model simulations. The vector ***e****_k_* = [0 … *d_k_* … 0]*^T^*, *k* = 1, …, *D*, is the perturbation size w.r.t. *k*th design parameter (*D* corresponds to the length of the ***z*** vector, i.e., the number of geometry parameters that represent the structure at hand) and ***d*** = [*d*_1_ … *d_k_* … *d_D_*]*^T^* is the vector of perturbations obtained for the structure under design. Note that, depending on the design stage, the vector of response features ***F****_f_* can be obtained from the responses of either ***C****_f_*, or ***B****_A_* models (cf. Section 2.2). Perturbations for construction of the low-fidelity-model-based Jacobian are set to 10^−3^.

In each iteration of (9), the gain ratio *ρ*—which represents actual change of the objective function obtained for the high-fidelity model versus the one predicted by the Taylor-expansion model—is calculated as [44]:

(12) $$\rho =\frac{U\left(F_{f}\left(z^{\left(j+1\right)}\right)\right)-U\left(F_{f}\left(z^{\left(j\right)}\right)\right)}{U\left(G^{\left(j\right)}\left(z^{\left(j+1\right)}\right)\right)-U\left(G^{\left(j\right)}\left(z^{\left(j\right)}\right)\right)}.$$

The gain ratio is used to assess quality of the solution obtained from (9). When *ρ* > 0, the design ***z***^(*j*+1)^ is accepted and used as a starting point for the next iteration. Otherwise, it is rejected and ***z***^(*j*)^ is re-used for optimization. The coefficient *ρ* is also used to update the TR radius *r*^(*j*)^ as follows,

(13) $$r^{\left(j+1\right)}=\{\begin{array}{l} \max\left(\eta_{1}\left\|z^{\left(j+1\right)}-z^{\left(j\right)}\right\|,r^{\left(j\right)}\right),\;\rho >0.9\\ \eta_{2}\left\|z^{\left(j+1\right)}-z^{\left(j\right)}\right\|,\;\rho <0.3\\ r^{\left(j\right)},\;\mathrm{otherwise} \end{array}$$

where the scaling factors for update of the TR radius are set to *η*_1_ = 2.5 and *η*_2_ = 0.25 [44]. The default initial radius is *r*^(0)^ = 1.

The algorithm is terminated when the obtained objective function value *U*(***F****_f_*(***z***^(*j*+1)^) is below 0 or when either of the following conditions is fulfilled: (i) the Euclidean distance between consecutive optimal designs, (ii) TR radius, or (iii) average change of objective function value for three consecutive iterations:

(14) $$\left\|z^{\left(j+1\right)}-z^{\left(j\right)}\right\|\leq \varepsilon$$

(15) $$r^{\left(j+1\right)}\leq \varepsilon$$

(16) $$\langle \sum_{i=j-1}^{j+1}\left|U\left(F_{f}\left(z^{\left(i-1\right)}\right)\right)-U\left(F_{f}\left(z^{\left(i\right)}\right)\right)\right|\rangle \leq \varepsilon .$$

Here, the user-defined threshold value is set to *ε* = 10^−2^. It should be noted that the numerical cost of (9) is only *D* + 1 evaluations of the structure per successful iteration. Additional simulations are required for *ρ* < 0.

The optimization algorithm can be summarized as follows:

(1) Set *j* = 0, ***z***^(*j*)^ = ***z***_0_, *r*^(*j*)^ = 1;
(2) Evaluate ***F****_f_*(***z***^(*j*)^) and select model ***F****_l_* = *P*(***R****_l_*) for construction of Jacobian;
(3) (Optional) Perform cell-level PE at ***z***^(*j*)^ as described in Section 2.5; select model ***F****_s_* = *P*(***R****_s_*) for Jacobian construction;
(4) (Optional) If *j* = 0, estimate *r*^(*j*)^ as explained in Section 3.3;
(5) Generate perturbations around ***z***^(*j*)^, construct the Jacobian and the ***G***^(*j*)^ model;
(6) Solve (9) to obtain a temporary solution ***z***_tmp_;
(7) Evaluate ***F****_f_*(***z***_tmp_), calculate *ρ* as in (12) and adjust radius *r*^(*j*+1)^ as in (13);
(8) If *ρ* > 0 set ***z***^(*j*+1)^ = ***z***_tmp_; otherwise set ***z***^(*j*+1)^ = ***z***^(*j*)^, *j* = *j* + 1 and go to Step 6;
(9) If *U*(***F****_f_*(***z***^(*j*+1)^) < 0 or either of conditions (14)–(16) is satisfied then END; otherwise set *j* = *j* + 1 and go to Step 2.

## 3. Automated Surrogate-Assisted Design of Compact Branch-Line Couplers

This section describes the proposed framework for automated surrogate-assisted design of couplers. In particular it provides a detailed discussion of each design step, i.e., cell-level optimization, two-stage design of low-fidelity BLCs, and surrogate-assisted tuning of high-fidelity coupler model. Each of the mentioned steps build up on the methods and algorithms of Section 2. The last part of the section contains summary of the framework. An important remark is that the main prerequisite for application of the presented framework to unsupervised design of couplers is availability of a feasible user-defined specifications (e.g., center frequency, bandwidth, power-split ratio, etc.) that are required to determine electrical parameters of individual cells used for coupler construction [32], as well as for controlling the objective functions. It should be reiterated that, to ensure low computational cost, each step of the design process that involves optimization is performed using a gradient algorithm embedded in the TR framework.

### 3.1. Optimization of the Pre-Defined Cells

As already mentioned, the conventional BLC comprises two pairs of horizontal and vertical TL sections with electrical length *θ*_0_ and characteristic impedances of *Z_H_*, and *Z_V_*, respectively. TLs parameters, that are appropriate for obtaining the desired coupler performance, can be determined based on the transmission line theory [58,65,66]. 

In the first design step, optimization of pre-defined CCs is performed. The process is oriented towards searching for the electrical properties of TLs that would normally be used for construction of conventional coupler. The feature-based objective function is given as:

(17) $$U_{C}\left(F_{f}(x)\right)=\max\left(l_{3}-l_{3\max},0\right)+\max\left(\left|l_{4}-\theta_{0}\right|-l_{4\max},0\right)+\left\|\begin{array}{c} \omega_{1}-\omega_{0}\\ \omega_{2}-\omega_{0} \end{array}\right\|.$$

As shown in Figure 6, *ω*_1_ and *ω*_2_ represent frequency points at which reflection of the cell has its minimal value and where the phase shift is equal to the desired value *θ*_0_, respectively. The coordinates *l*_3_ and *l*_4_ represent the reflection and phase shift at the center frequency *ω*_0_. The user-defined parameters *l*_3max_ = 0.003 and *l*_4max_ = 0.1° are used to activate/deactivate the first two components of (17). Note that cells optimization is performed at the low-fidelity model level (i.e., *f* = *l*; cf. Section 2.2). The feature-based response is calculated individually for each cell, i.e., ***F****_f_*(***x***) = ***F****_f.V_*(***x***) = *P*(***C****_f_*_.*V*_(***x***_o_)), or ***F****_f_*(***x***) = ***F****_f.H_*(***x***) = *P*(***C****_f_*_.*H*_(***x***_o_)). 

The starting point for cell-level optimization is selected from a set of random samples generated as follows:

(18) $$X=\left\{x|x=\left(u_{b}-l_{b}\right)\circ y+l_{b},y\in R^{D},0\leq y\leq 1\right\}.$$

Here, ***l****_b_* and ***u****_b_* represent the lower and upper bounds on geometry parameters of the cell at hand. The symbol “○” represents component-wise multiplication. The starting point for cell optimization is obtained from:

(19) $$x_{\mathrm{init}}=\arg\underset{x\in X}{\min}\left(U_{C}\left(F_{l}(x)\right)\right).$$

In other words, ***x***_init_ is the design from the set ***X*** for which the objective function (17) has the lowest value. The cell is optimized by solving (9). Due to utilization of equivalent-circuit model representation, the cost of finding the initial and optimal solutions is negligible. Finally, the parameters of the optimized cells ***x***_H_* and ***x***_V_* (cf. Section 2.2) are concatenated and used as a starting point for coupler design.

It should be emphasized that the role of cell-level optimization is twofold. On one hand, the procedure rejects the cells that are incapable of fulfilling the user-defined specifications. On the other hand, it allows to find optimal design parameters of selected CCs.

### 3.2. Two-Stage BLC Optimization

Upon completion of selection and optimization procedure, the CCs are used to construct the BLC model (cf. Section 2.2) which then undergoes a two-stage design process. In the first step, the coupler is optimized w.r.t. performance requirements. The design criteria include: (i) maximization of bandwidth, (ii) maintaining minimum of *S*_11_ and *S*_41_ at *ω*_0_, and (iii) minimization of power-split error at ω_0_. The objective function is defined as,

(20) $$\begin{array}{c} U_{B.1}\left(F(y)\right)=\beta_{1}\left(-BW+B_{0}\right)+\left\|\begin{array}{c} \omega_{5}-\omega_{0}\\ \omega_{6}-\omega_{0}\\ \omega_{7}-\omega_{0} \end{array}\right\|+{\left(\frac{\max\left(\left|\Delta C-\Delta C_{0}\right|-\Delta C_{\max},0\right)}{\Delta C_{\max}}\right)}^{2}+\\ +\beta_{2}{\left(\frac{\max\left(\left\{l_{5},l_{6}\right\}-S_{\max},0\right)}{S_{\max}}\right)}^{2} \end{array}$$

where ***F***(***y***) = *P*(***B****_f_*(***y***)). The feature coordinates *ω_n_* and *l_n_*, *n* = 1, 2, …, 9 are defined as shown in Figure 6. The parameters *ω_l_* = max(*ω*_1_, *ω*_2_) and *ω_h_* = min(*ω*_3_, *ω*_4_) represent lower and upper corner frequency for which |*S*_11_| and |*S*_41_| are both below the specified threshold (here, −20 dB). The bandwidth is defined as *BW* = 2min{*ω_h_* − *ω_0_*, *ω_0_* − *ω_l_*,}. Parameters Δ*C*_0_, Δ*C*_max_, and *S*_max_ denote desired power-split imbalance at the center frequency, acceptable power-split error—i.e., deviation of Δ*C* = |*l*_8_ − *l*_9_| from the desired value of Δ*C*_0_ (here, 0.2 dB)—and maximum allowed reflection level of *S*_11_ and *S*_41_ at *ω*_0_ (here, −26 dB). The scaling coefficients are set to *β*_1_ = 10 and *β*_2_ = 1000. The particular values are determined based on the numerical experiments.

The starting point for performance-wise coupler optimization is ***y***_0_ = [***x***_H_* ***x***_V_*]*^T^* (cf. Section 3.1). The final design is found through minimization of (20) by solving (9). The obtained solution ***y***_1_* is set as the starting point for a second-stage of the low-fidelity-model-based BLC design, i.e., minimization of coupler size. The objective function is given by:

(21) $$U_{B.2}\left(F(y)\right)=A_{B}\left(y\right)+\beta {\left(\max\left(U_{B.1},0\right)\right)}^{2}.$$

Here, *β* = 10. Due to representation of the BLC using equivalent-circuit model, the cost of minimizing (20)–(21) is low. The final design (here, denoted as ***y***_2_*) is used as a starting point for surrogate-assisted optimization of the assembled coupler, as described below.

### 3.3. Surrogate-Assisted Optimization

The final design stage involves surrogate-assisted optimization of the assembled coupler. In each SBO iteration, the feature-based linear model (10) is constructed from a single EM response of the ***B****_A_* model that represents the assembled BLC. The Jacobian ***J*** is calculated based on the responses of the BLC surrogate model (*f* = *s*). In other words, in (10), ***F****_f_*(***z***^(*i*)^) is calculated based on the ***B****_A_* response obtained at design ***z***^(*i*)^, whereas ***J***(***z***^(*i*)^) is constructed from ***B****_s_* simulations—determined based on corrected responses of compact cells—obtained around the ***z***^(*i*)^ design. The surrogate models of horizontal and vertical cells that constitute the ***B****_s_* model are individually corrected to the high-fidelity model level using implicit SM and frequency scaling (cf. Section 2.4). The rationale behind correction of individual cells is that PE can be carried out using relatively small number of control parameters, which is beneficial for fast convergence of TR-based optimization. Furthermore, the BLC surrogate, composed of individually corrected CCs, is characterized by good generalization capability [54].

At the beginning of SBO, the initial TR radius is estimated. The proposed estimation process is realized in a separate TR loop that exploits linear approximation model ***G*** constructed from ***B****_s_* responses. The algorithm can be summarized as follows:

(1) Set *j* = 0, *r*_tmp_^(0)^ = 0.5;
(2) Set ***y***^(*j*)^ = ***y***_2_*;
(3) Find ***y***^(*j*+1)^ through minimization of the objective function (20) by solving (9);
(4) Evaluate ***F****_s_*(***y***^(*j*+1)^) = *P*(***B****_s_*(***y***^(*j*+1)^)) and calculate *ρ*;
(5) If *ρ* > 0.5, set *r*_tmp_^(*j*+1)^ = 0.5(*r*_tmp_^(*j*)^ + 1); otherwise set *r*_tmp_^(*j*+1)^ = 0.5(*r*_tmp_^(*j*)^ + 0.1);
(6) If *j* = 5 set *r*^(0)^ = *r*_tmp_^(*j*+1)^ and exit; otherwise set *j* = *j* + 1 and go to Step 2.

It should be reiterated that the surrogate model ***B****_s_* does not account for cross-couplings effects between the cells and therefore exhibits a certain level of inaccuracy (most notably in the form of frequency shift, or power-split discrepancy w.r.t. the ***B****_A_* model). This is accounted for through refinement of the estimated radius as *r*^(0)^ = *r*^(0)^*s_F_*, where *s_F_* is the scaling factor that averages the discrepancy between *ω*_5_, *ω*_6_ features obtained for the surrogate and fine model. The optimization process is oriented towards minimization of (20) using the version of the TR algorithm that employs both optional steps (cf. Section 2.6). Moreover, additional constraint is introduced to (9) in order to ensure that the footprint of optimized BLC does not exceed *A_B_*(***y***_2_*) (cf. Section 2.2 and Section 3.2). The vector y* obtained after completing SBO is the final solution of unsupervised design process.

### 3.4. Summary of the Design Framework

The proposed design framework can be summarized as follows:

(1) Define coupler-level specifications and determine the electrical parameters of individual BLC sections that correspond to the design requirements;
(2) Find the starting point for cell-level design as described in Section 3.1;
(3) Minimize (17) to select and optimize CCs that are capable of fulfilling the imposed specifications;
(4) Use the selected CCs for construction of the ***B****_c_* model. Sequentially minimize (20) and (21) to obtain ***y***_1_* and ***y***_2_*;
(5) Perform SBO using ***B****_A_* and ***B****_s_* models to obtain the final BLC design y* (cf. Section 3.3).

The user-defined figures for automated design include electrical length of cells, center frequency, power-split imbalance, bandwidth, and substrate properties. It should be noted that characteristic impedances of CCs required to obtain selected Δ*C*_0_ can be calculated from [65],

(22) $$Z_{H}=Z_{0}\sqrt{C_{0}/\left(1+C_{0}\right)},\;Z_{V}=Z_{0}\sqrt{C_{0}}$$

where *C*_0_ = 10^Δ*C*0/10^ and *Z*_0_ = 50 Ω. Note that for power-split imbalance Δ*C*_0_ = 0, the BLC features equal (3-dB) power-split. It should be reiterated that, owing to embedding each design step (including PE of cells) into the TR loop, the overall cost of coupler design using the proposed framework is low. Successful iterations of surrogate-assisted BLC refinement require only one EM simulation per CC (for PE) and one EM evaluation of the assembled coupler. Unsuccessful steps require additional simulations of ***B****_A_* (PE is performed only for successful iterations).

## 4. Numerical Results

The main focus of this section is description of the library of pre-defined cells followed, as well as numerical validation of the proposed design framework. The methodology is demonstrated based on three examples concerning (i) design of a compact coupler with unequal power-split, (ii) comparison of BLCs characterized by different topologies w.r.t. size/performance trade-off, and (iii) rapid re-design of structures for various substrate parameters. The section is summarized by the discussion and comparison of the proposed framework against the methods from the literature. It should be noted that all numerical experiments have been performed on a dual Intel Xeon E5540 machine with 32 GB of RAM. Moreover, unless stated otherwise, the dimensions of all structures considered in this section are expressed in mm.

### 4.1. Database of Compact Cells

**Figure 9:**
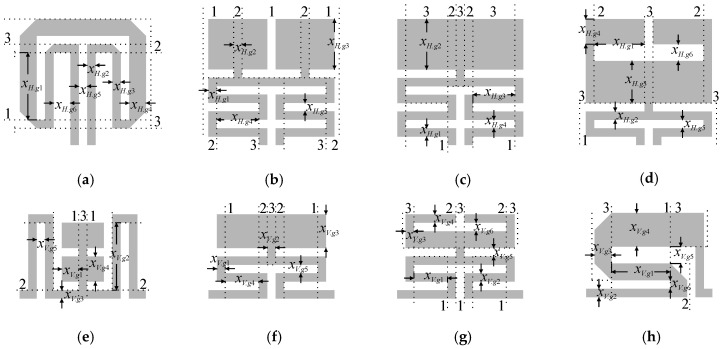
The database of compact cells used for automated BLC design. Dotted lines represent sub-sections of the substrate characterized by different permittivity/thickness pairs—each index *k* = 1, 2, 3, corresponds to *ε_rk_*/*h_k_* of the given sub-section. Four horizontal (**a**) ***C****_H_*^(1)^, (**b**) ***C****_H_* ^(2)^, (**c**) ***C****_H_* ^(3)^, (**d**) ***C****_H_* ^(4)^ and four vertical (**e**) ***C****_V_*^(1)^, (**f**) ***C****_V_* ^(2)^, (**g**) ***C****_V_* ^(3)^, (**h**) ***C****_V_* ^(4)^ cells are implemented. The structures are designed in the form of an equivalent-circuit and EM models.

The topologies of the CCs used in this work are shown in Figure 9. The library contains four horizontal ***C****_H_*^(*i*)^, *i* = 1, 2, 3, 4, and four vertical ***C****_V_*^(*j*)^, *j* = 1, 2, 3, 4, cells. Each is implemented at the level of both equivalent-circuit and EM simulation models. As already mentioned in Section 2, the cells are described using a set of geometry variables ***x****_o_* = [*x_o.g_*_1_ *x_o.g_*_2_ …]*^T^*, setup parameters ***s****_o_*, and dimension *x*_gap_ that represents distance between cells embedded into the BLC circuit. When necessary, the cells also implement the correction parameters ***p*** = [***x****_c_*^(*j*)^ ***α***^(*j*)^]*^T^*. The meaning of all mentioned variables as well as their role within the cell model is illustrated in Figure 5. Apart from containing information on cell orientation, the database encompasses ranges of design parameters for which CC geometries remain feasible, functions for calculating cells dimensions, etc. For simplicity of notation, the coupler composed of *i*th horizontal and *j*th vertical cell is denoted as ***B***^(*i*,*j*)^. The high-fidelity EM models and low-fidelity equivalent-circuit models of cells are implemented in Keysight ADS [67].

### 4.2. Design of Unequal-Split BLC

The first example is the design of an unequal-power-split coupler implemented on a Rogers RO4003C substrate (*ε_r_* = 3.38, *h* = 0.813 mm, tan*δ* = 0.0021). The following design specifications are considered: center frequency *ω*_0_ = 1.5 GHz, power-split imbalance Δ*C*_0_ = 3 dB, and bandwidth *BW* ≥ 150 MHz. The coupler is composed of cells ***C****_H_*^(4)^ and ***C****_V_*^(3)^ (cf. Figure 9). Their characteristic impedances—calculated from (22)—are *Z_H_* = 40.8 Ω and *Z_V_* = 70.6 Ω, respectively. The gap is set to *x*_gap_ = 0.3 mm.

**Figure 10:**
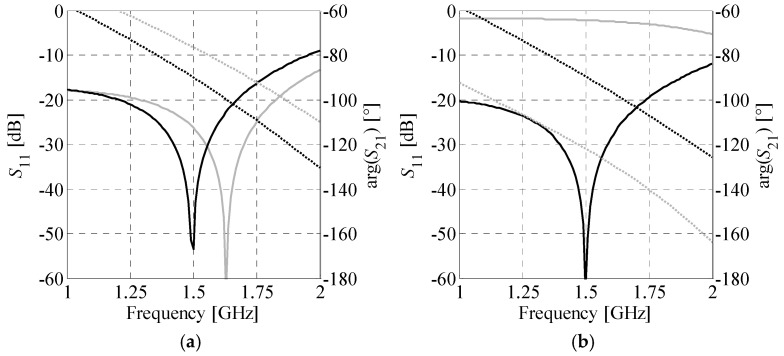
Low-fidelity model reflection (–) and phase (···) responses of individual cells at the randomly generated initial (gray) and optimized (black) designs: (**a**) cell *C_H_*^(4)^ and (**b**) cell *C_V_*^(3)^.

**Figure 11:**
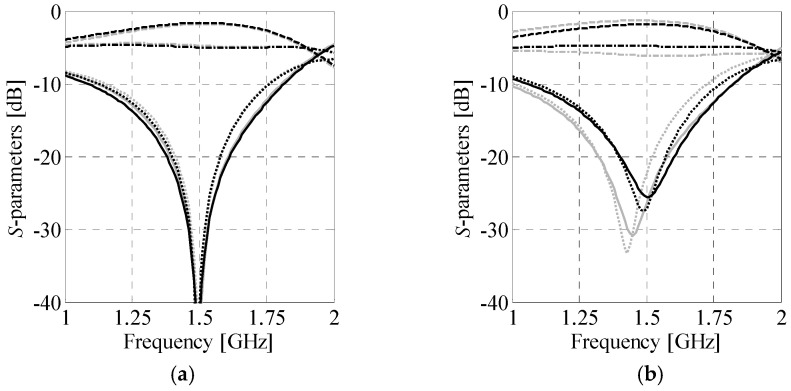
BLC responses vs. frequency at various stages of the design process: (**a**) low-fidelity model-based characteristics at ***y***_0_ (gray) and ***y***_2_* (black) and (**b**) EM-based responses of assembled coupler at ***y***_2_* (gray) and y* (black). The characteristics are marked as follows: *S*_11_ (–), *S*_21_ (– –), *S*_31_ (–·), *S*_41_ (···).

The structure was designed according to the methodology outlined in Section 3.4. In the first step, the starting points for CCs optimization were obtained using procedure of Section 3.1 and the designs ***x***_H_* = [2.49 0.41 2.29 0.49 0.66 0.48]*^T^* and ***x***_V_* = [3.38 0.2 0.2 0.82 1 1]*^T^* were found through minimization of (17). Comparison of cells responses at the initial and optimized designs is shown in Figure 10. Next, a two-stage optimization of the coupler was performed as described in Section 3.2. The initial design was set to ***y***_0_ = [***x***_H_* ***x***_V_*]*^T^*. The design ***y***_1_* = [2.6 0.37 1.44 2.16 0.9 0.28 3.71 0.2 0.28 0.39 1 0.98]*^T^*, obtained by minimization of (20) using (9), was set as a starting point for size-oriented coupler optimization. The final design ***y***_2_* = [2.69 0.33 1.45 2.16 0.9 0.28 3.64 0.2 0.25 0.36 0.99 0.98]*^T^* was found through minimization of (21). The coupler footprints at ***y***_0_, ***y***_1_* and ***y***_2_* are 295 mm^2^, 285 mm^2^, and 276 mm^2^, respectively. Low-fidelity model responses of the structure at ***y***_0_ and ***y***_2_* designs are compared in Figure 11a. It should be noted that the geometry obtained directly from optimization of individual CCs already offers acceptable characteristics. The two stage optimization further improved the performance and reduced the BLC footprint. In the last step, surrogate-based optimization of the assembled coupler ***B****_A_* that accounts for cross-couplings between individual cells—was performed as described in Section 3.3. The high-fidelity optimized design y* = [1.97 0.29 1.68 2.31 0.84 0.39 3.98 0.36 0.34 0.2 0.78 0.9]*^T^* was found after only four SBO iterations. Responses of the high-fidelity EM model at ***y***_2_* and y* designs are compared in Figure 11b. The dimensions of the optimized structure are 10 mm × 28 mm = 280 mm^2^, which corresponds to 75.5% size reduction compared to conventional BLC coupler (dimensions: 31.8 mm × 36 mm = 1144.8 mm^2^). 

**Figure 12:**
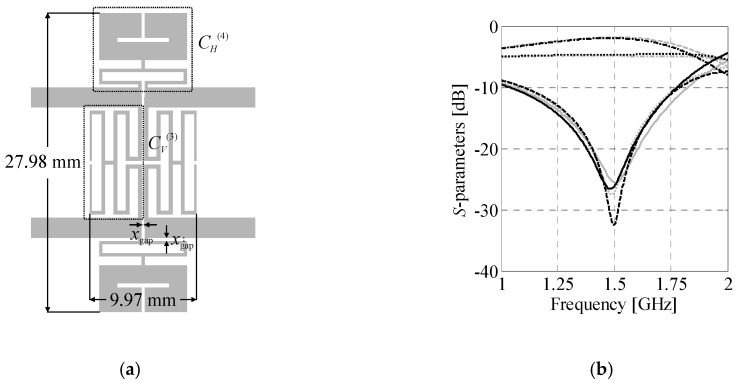
Optimized BLC: (**a**) geometry and (**b**) comparison of the high-fidelity EM model ***B****_A_* responses: *S*_11_ (–), *S*_21_ (– –), *S*_31_ (–·), *S*_41_ (···) obtained using Keysight ADS (gray) and CST Studio (black).

Geometry of the optimized structure, as well as comparison of the BLC responses obtained from EM simulations performed using Keysight Momentum and CST Microwave Studio (time-domain solver) [68] are shown in Figure 12. The results are in good agreement. It should be reiterated that the coupler design was performed without user supervision. The overall computational cost of BLC design corresponds to just 12.1 evaluations of the ***B****_A_* model (around 11 min of CPU-time) including 4 ***B****_A_* simulations, a total of 6 EM simulations of ***C****_H.h_*^(4)^ and ***C****_V.h_*^(3)^ required for SBO refinement, as well as some computational overhead for PE and TR-based optimization.

The proposed design approach was benchmarked against the state-of-the-art SBO algorithms [44,48,62]. The considered methods include: (i) direct TR-based optimization of ***B****_A_* model, (ii) two versions of implicit SM executed at the level of ***B****_l_* model, (iii) two versions of ISM combined with frequency scaling, and (iv) the version of algorithm of Section 3 that does not exploit the mechanism for automated estimation of the initial radius at the beginning of SBO. The figures considered for comparison include optimization cost, as well as bandwidth, power-split, phase-shift, and size of the final designs. For fair comparison, the design ***y***_2_* was set as a starting point for each benchmark algorithm. The results shown in Table 1 indicate that ISM-based routines failed to obtain satisfactory solutions due to divergence. Algorithm (i) yields the best results in terms of structure size and bandwidth. The designs obtained using (iii) are characterized by the largest footprints and highest deviations of Δ*C* from the target value. The proposed design approach yields relatively small design while ensuring decent electrical performance. It should be noted that the bandwidth of designs obtained using benchmark algorithms is broader compared to the one determined using the proposed algorithm. However, they also feature worsened power-split imbalance. The reason is that the objective function used for BLC optimization aggregates performance-related design objectives into a weighted sum [45,69]. From the objective function standpoint, bandwidth enhancement compensates the degradation of power-split imbalance.

The proposed design optimization approach provides the lowest computational cost among algorithms that yield acceptable designs. For the considered example, the routine of Section 3 is about 85% faster compared to (i), but also its cost is up to 82% lower compared to methods based on algorithm (iii). Moreover, the proposed algorithm is over 32% faster compared to the method that does not involve automatic determination of the initial TR radius. It should be emphasized that significant computational savings w.r.t. algorithms (i)–(iii) result from embedding the parameter extraction process into the TR framework, which dramatically reduces the number of required simulations. As can be seen from Table 1, this change reduces the number of low-fidelity model evaluations required for SBO by one to two orders of magnitude. Another advantage of the proposed algorithm is that parameter extraction is performed at the level of individual cells of the coupler, which improves generalization of the BLC surrogate compared to (i)–(iii).

### 4.3. Size/Performance Comparison of Compact BLCs

The second example concerns comparison of compact BLCs with respect to their performance and size. A total of 16 topologies, representing all combinations of cells from the library of Section 4.1, are used for analysis. The design specifications are as follows. The couplers are to be implemented on a Rogers RO4003C substrate (*ε_r_* = 3.38, *h* = 0.813 mm, tan*δ* = 0.0021). 

The performance-related design objectives include center frequency of 1 GHz, equal-power split, and at least 90 MHz bandwidth. Based on (22), the equal-split (Δ*C*_0_ = 0 dB) is obtained for *Z_V_* = 50 Ω and *Z_H_* = 35.4 Ω, respectively. The gap between the adjacent cells is fixed to *x*_gap_ = 0.3 mm. First, all CCs from the database were optimized as described in Section 3.1. Due to insufficient performance, the cell *C_V_*^(3)^ was rejected by the algorithm from the design process. Next, a set of 12 coupler topologies composed of ***C****_H_*^(*j*)^, *i* ∈ {1, 2, 3, 4}, horizontal and ***C****_V_*^(*j*)^, *j* ∈ {1, 2, 4}, vertical cells that feature satisfactory performance was selected for two-stage optimization. Finally, the resulting BLC designs were refined to the high-fidelity model level using surrogate-assisted optimization. 

**Figure 13:**
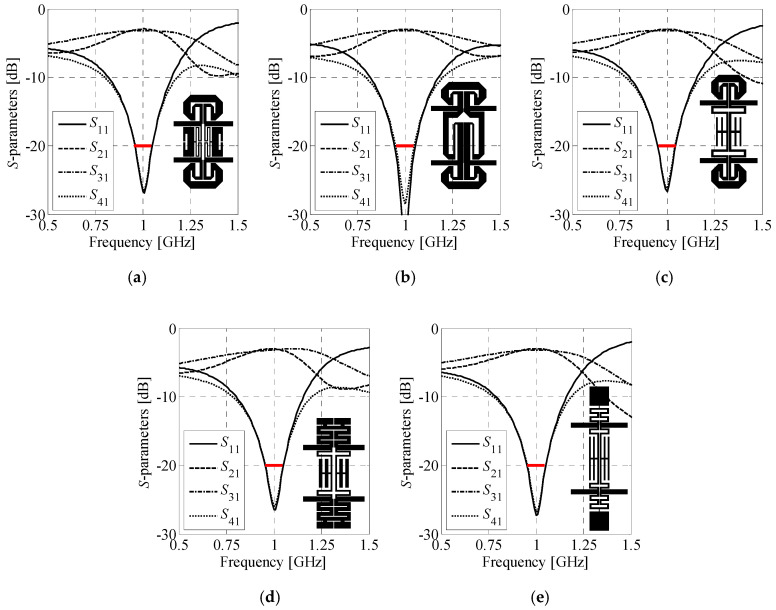
Frequency responses and geometries (in-scale) of optimized couplers (high-fidelity EM models) featuring acceptable performance: (**a**) ***B****_A_*^(1,2)^, (**b**) ***B****_A_*^(1,4)^, (**c**) ***B****_A_*^(1,1)^, (**d**) ***B****_A_*^(2,1)^, and (**e**) ***B****_A_*^(3,1)^.

Table 2 shows comparison of the couplers in terms of bandwidth, power split imbalance, phase shift, size, and miniaturization. To provide more unequivocal measure of structures properties, a performance-to-size coefficient (*PtS*) was defined as,

(23) $$PtS^{\left(i,j\right)}=\left(BW^{\left(i,j\right)}-200\max{\left(\frac{\Delta C^{\left(i,j\right)}-\Delta C_{\max}}{\Delta C_{\max}},0\right)}^{2}\right)\cdot \frac{1}{0.1A_{B}{}^{\left(i,j\right)}}$$

where *BW*^(*i*,*j*)^, Δ*C*^(*i*,*j*)^ and *A_B_*^(*i*,*j*)^ denote bandwidth, power-split imbalance, and size of the optimized ***B****_A_*^(*i*,*j*)^ coupler. The parameter Δ*C*_max_ = 0.2 dB (cf. Section 3.2). Owing to being proportional to quality of the BLC with respect to design requirements, *PtS* can be used to sort BLCs w.r.t. their performance. Structures with negative *PtS* are considered unacceptable from the specification standpoint. The results of Table 2 indicate that only five of twelve couplers, i.e., ***B****_A_*^(1,2)^, ***B****_A_*^(1,4)^, ***B****_A_*^(1,1)^, ***B****_A_*^(2,1)^, and ***B****_A_*^(3,1)^, feature acceptable performance. The remaining ones are characterized by either too narrow bandwidth or unacceptably high power-split imbalance. It should be noted that even the designs with *PtS* > 0 may slightly violate requirement concerning Δ*C*_max_. As already mentioned, this is due to aggregation of objectives in (20) using weighted sum. Therefore, slight violation of the requirements can be considered acceptable. Frequency characteristics and geometries of couplers with satisfactory performance are shown in Figure 13, whereas their detailed dimensions are gathered in Table 3.

Another important remark is that the performance of the optimized BLCs vary significantly with topology. In other words, compact dimensions of a coupler often come at the expense of performance deterioration [26,70]. A satisfactory trade-off between the size and the performance of the BLC can be achieved, provided that appropriate combination of cells is selected. The results are on par with findings from the literature references, indicating that size- and performance-related objectives are conflicting [16,37,38].

The average design cost is only 18.2 min of CPU-time per coupler. The cost accounts for two-stage optimization of the assembled structure low-fidelity model (~3 min per design), and surrogate-assisted refinement to the EM model level. The overall design cost of 12 BLCs amounts to 3.4 hours. It should be reiterated that all the couplers were designed without external supervision.

### 4.4. BLC Re-Design for Substrates with Different Parameters

**Figure 14:**
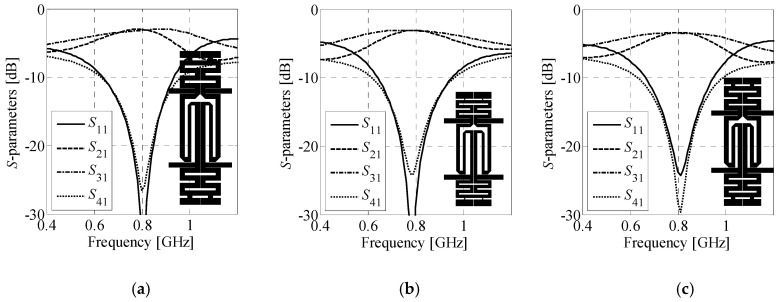
Frequency characteristics and geometries (in-scale) of the ***B****_A_*^(2,4)^ coupler re-designed to work on: (**a**) Arlon AD250C, (**b**) Arlon AD300C, and (**c**) FR-4 substrates.

The last example concerns re-design of the coupler for substrates with different parameters. The structure of choice consists of ***C****_H_*^(2)^ and ***C****_V_*^(4)^ cells. The gap between CCs is set to *x*_gap_ = 0.2 mm. Geometry of the coupler is shown in Figure 14. The design specifications are as follows: *ω*_0_ = 0.8 GHz, Δ*C*_0_ = 0 dB and *BW* ≥ 80 MHz. The structure is re-designed for operation on Arlon AD250C (*ε_r_* = 2.5, *h* = 0.762 mm, tan*δ* = 0.0014), Arlon AD300C (*ε_r_* = 2.97, *h* = 0.762 mm, tan*δ* = 0.002), and FR-4 (*ε_r_* = 4.3, *h* = 1 mm, tan*δ* = 0.02) substrates, respectively. 

For each substrate the coupler was designed using the framework of Section 3. Dimensions of the optimized structures and their performance figures are gathered in Table 4 and Table 5, respectively. Figure 14 shows frequency responses of the obtained designs. Differences in dimensions of the individual designs indicate that the couplers cannot be accurately scaled using transmission line theory methods [71]. Despite similar performance characteristics, two of the optimized designs slightly violate the requirements concerning bandwidth. The obtained results are considered acceptable (especially having in mind complexity of the objective function used for their optimization). The average computational cost of BLC re-design is around 17.4 min of CPU-time.

### 4.5. Discussion and Measurements

The proposed design framework employs several mechanisms that make it unique compared to other database-inspired solutions considered in the literature [16,32,37,72]. First of all, it reduces the involvement of the user in the design process, merely to choose the specifications and substrate parameters. Moreover, due to integration of design steps into the TR loop, the method generates optimal designs at a cost that is substantially lower than conventional SBO approaches [32,53,54]. The difference is especially pronounced when high-fidelity EM models, used for evaluation of structure performance are efficient [53]. Finally, the algorithm supports adjustment of substrate parameters, and automated generation of the initial designs for the cell-level optimization.

As indicated in Table 6, the proposed framework provides improvement in terms of computational cost, flexibility of operation and design automation as compared to other algorithms based on the concept of re-using cells from the library [16,32,72]. For instance, in [16], the cells are represented only at the level of EM models, which negatively affects the cost of their tuning. Moreover, the method does not permit changing cell properties (substrate parameters, input impedance), which limits its flexibility in terms of cells application scenarios. Finally, the method in [16] is, at best, semi-automatic as it does not provide standardized mechanisms for cells/couplers tuning. In [72], a relatively low design cost is maintained using surrogate-assisted optimization. The method, however, does not provide mechanisms for rejection of structures with inferior performance. Instead, it yields a set of designs representing trade-off between selected performance figures to compare miniaturized circuits. On the other hand, in real-life one is normally interested in finding a single design that meets the specifications. In this regard, the redundancy of the generated solutions is undesirable from the standpoint of computational cost unless designer’s priorities are not clearly defined [45,72]. In [32], a methodology dedicated to design bandwidth-enhanced couplers is presented. Although the method offers mechanisms for adjusting the cells for broadband operation, their selection is still driven by the user. Furthermore, the method is expensive in terms of CPU-time due to application of numerically inefficient EM representation of coupler.

Despite using pre-defined cells, the results of Section 4.3 show that the proposed framework is capable of producing designs that are competitive (miniaturization-wise) even with manually developed BLCs. Table 7 provides benchmark of the coupler ***B****_A_*^(3,1)^ against compact BLCs from the literature. For fair comparison, dimensions of structures have been expressed in terms of a guided wavelength *λ_g_* calculated for the given center frequency and substrate parameters. Moreover, the bandwidth *BW*—expressed in percent to account for different operating frequencies of considered circuits—is defined for |*S*_11_| and |*S*_41_| being both below −20 dB. The structure of Section 4.3 offers not only substantial miniaturization rate of almost 80% but also maintains nearly 10% bandwidth, which is more than for majority of considered BLCs. It should be noted that the couplers of [18,20] have been miniaturized using fractal curves. For considered operation frequencies, practical application of fractals is limited only to thin substrate materials (*h* < 0.2 mm). The reason is that thin substrates are required to obtain relatively narrow low-impedance TLs, which can be folded by more than one iteration of the selected fractal shape.

As shown in Section 4.4, the proposed methodology can also be used for rapid re-design of BLCs w.r.t. given substrate parameters. Although, the techniques for scaling microwave and antenna structures at a cost corresponding to only a few EM simulations have been reported in the literature [71], the prerequisite for their application is availability of an inverse model. The latter is identified based on a set of individually optimized training points. Since the number of reference designs grows quickly with the number of figures being of interest from the structure re-design standpoint, construction of inverse surrogate might be impractical when it is not intended for multiple re-use. In this work, re-design of the structure for different substrates was performed at an average cost corresponding to about 19 high-fidelity EM model simulations (~17 min of CPU-time) which is low having in mind that, in [71], up to 13 independently optimized reference designs were used for constructing inverse surrogate dedicated for scaling of couplers w.r.t. only one figure of interest.

**Figure 15:**
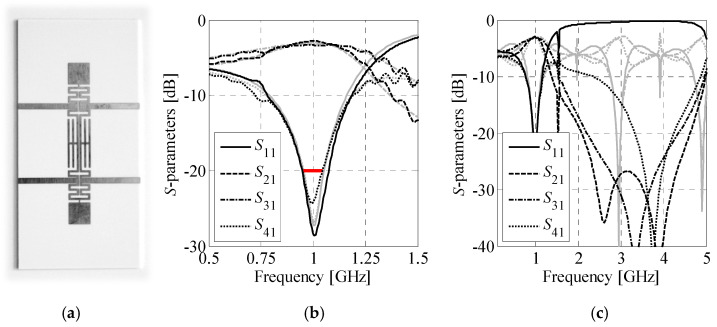
Branch-line coupler ***B****_A_*^(3,1)^ of Section 4.3: (**a**) photograph of the manufactured circuit prototype, (**b**) comparison of simulated (gray) and measured (black) responses, and (**c**) comparison of the broadband frequency characteristics (black) against the responses of conventional BLC (gray).

The coupler ***B****_A_*^(3,1)^ of Section 4.3 has been fabricated and measured. Photograph of the manufactured structure is shown in Figure 15a, whereas comparison of simulation and measurement results is given in Figure 15b. The characteristics are in good agreement and the bandwidths are similar. Slight discrepancies between the obtained responses result from fabrication tolerances and the circuit assembly components which were not accounted for in the simulation models. 

It is worth noting that low-pass properties of compact cells make them useful for construction of BLCs with harmonic suppression capability [15,24,28,31,32]. The mechanism is demonstrated in Figure 15a where broadband responses of the ***B****_A_*^(3,1)^ structure are compared against characteristics of the conventional BLC. Although the CCs used for construction of ***B****_A_*^(3,1)^ circuit have been optimized only for the center frequency of 1 GHz (cf. Section 3 and Section 4.3), the coupler features |*S*_21_| and |*S*_31_| below −20 dB within 2.2 GHz to 4.3 GHz range. At the same time, the conventional structure is not capable of suppressing unwanted frequencies. 

In summary, the obtained numerical results indicate that the proposed framework can be used not only for unsupervised design of BLCs, but also for the comparison of compact topologies, and re-purposing the circuits for various substrates. Re-implementation of the SBO mechanisms around the TR-based algorithm ensures substantial reduction of optimization cost, as compared to conventional surrogate-assisted routines. The proposed mechanisms might be useful for design of other—more complex—structures with modular topology. Finally, the measurement results confirm the correctness of the used EM simulation models, whereas broadband simulations indicate the capability of the considered structures in suppressing harmonic frequencies.

## 5. Conclusions

A framework for low-cost automated design of compact branch-line couplers has been presented. The proposed methodology is based on a bottom-up design concept that involves automated selection of cells, bi-stage optimization of assembled BLC with respect to performance and size, and surrogate-assisted refinement of the coupler to the high-fidelity model level. To ensure the low operation cost, each step of the design process is embedded in a trust-region framework. Furthermore, the framework exploits feature-based representation of structure responses—at cell- and coupler-levels—and space-mapping-correction of low-fidelity models responses. It should be emphasized that the high-fidelity EM representation of BLC is used only during the final stage of the design process.

The performance of the proposed framework was demonstrated using three test cases concerning rapid optimization of compact coupler, size-oriented comparison of BLC geometries, and rapid re-design of coupler for various substrates. The average design cost using the presented methodology amounts to just 17.5 min of CPU-time. The approach proved to be useful for generating topologies featuring miniaturization rates of nearly 80%. The numerical performance and quality of the obtained designs have been positively benchmarked against other routines from the literature. The selected simulation results have been confirmed by measurements of fabricated BLC prototype.

Although the proposed methodology has been demonstrated only on BLC couplers, it is suitable for variety of circuits characterized by modular topologies such as antenna feeding networks, multi-band or broadband couplers, and others. Further work will focus on the adaptation of the framework for design of mentioned components.

## Figures and Tables

**Table 1:** Surrogate-Assisted BLC Design: Benchmark Results.

Design Approach *	Number of Evaluations	Cost		*BW* [MHz]	Δ*C* @*ω*_0_ [dB]	∠(*S*_21_/*S*_31_) @*ω*_0_ [°]	Size [mm^2^]
		*B_A_* (min)	Total [min]				
**(i)**	**84 *B_A_***	**84 (77)**	**77**	**190**	3.14	89.9	266.8
(ii)^1,#^	1240 *B_l_* 2 *B_A_*	36.1 (33.1) 2 (1.8)	34.9	N/A	N/A	N/A	N/A
(ii) ^2,#^	2749 *B_l_* 6 *B_A_*	80 (73.3) 6 (5.5)	78.8	N/A	N/A	N/A	N/A
(iii) ^1^	2144 *B_l_* 5 *B_A_*	62.4 (57.2) 5 (4.6)	61.8	180	2.75	90.1	282.6
(iii) ^2,*$*^	1371 *B_l_* 3 *B_A_*	39.8 (36.6) 3 (2.8)	39.4	190	3.28	90.1	299.7
(iv)	296 *C_l_* 8 *C_h_* 55 *B_l_* 7 *B_A_*	4.3 (3.9) 5.1 (4.7) 1.6 (1.5) 7 (6.4)	16.5	180	3.23	90.2	276.5
This work	221 *C_l_* 6 *C_h_* 39 *B_l_* 4 *B_A_*	3.2 (3.0) 3.8 (3.5) 1.1 (1.0) 4 (3.7)	11.2	170	3.02	90.3	276.5

* Initial design is set to ***y***_2_*; average cost of obtaining ***y***_2_* using method of Section 3 is ~3 min. ^1^ 8 preassigned parameters that represent substrate thickness. ^2^ 16 preassigned parameters that represent substrate thickness and permittivity. ^#^ Terminated due to divergence. ^$^ Terminated due to divergence after yielding quasi-optimal solution.

**Table 2:** Performance comparison of optimized compact BLCs.

Coupler Topology	*BW* [MHz]	Δ*C* @*ω*_0_ [dB]	∠(*S*_21_/*S*_31_) @*ω*_0_ [°]	Dimensions [mm × mm]	Size [mm^2^]	Size Reduction [%] *	*PtS ^#^*
*B_A_* ^(3,2)^	76	0.66	90.5	10.0 × 36.8	369	82.1	−26.0
*B_A_* ^(4,1)^	45	0.50	90.1	21.6 × 25.7	554	73.1	−7.47
*B_A_* ^(3,4)^	89	0.44	87.7	9.31 × 43.3	403	80.5	−4.99
*B_A_* ^(2,2)^	10	0.40	90.0	14.1 × 30.6	432	79.0	−4.33
*B_A_* ^(2,4)^	56	0.40	92.3	12.1 × 40.6	495	76.0	−3.02
*B_A_* ^(4,2)^	20	0.33	90.2	17.5 × 23.7	414	79.9	−1.66
*B_A_* ^(4,4)^	89	0.35	89.3	20.8 × 31.0	644	68.8	−0.28
*B_A_* ^(1,2)^	91	0.28	89.9	14.9 × 35.2	524	74.6	1.09
*B_A_* ^(1,4)^	100	0.21	87.1	17.2 × 40.2	692	66.4	1.44
*B_A_* ^(1,1)^	94	0.23	90.1	13.3 × 42.9	573	72.2	1.55
*B_A_* ^(2,1)^	96	0.15	89.9	12.8 × 41.5	533	74.1	1.80
*B_A_* ^(3,1)^	99	0.24	90.1	7.72 × 54.6	421	79.6	2.20

* Calculated w.r.t. conventional 3dB (Δ*C* = 0) BLC operating at *ω*_0_ = 1 GHz and implemented on RO4003C with dimensions of 45.9 mm × 44.9 mm (footprint of 2061 mm^2^). ^#^ Only couplers with *PtS* > 0 feature acceptable performance w.r.t. specifications.

**Table 3:** Dimensions of couplers featuring acceptable performance.

Coupler Topology	*x_H_*_.1_	*x_H_*_.2_	*x_H_*_.3_	*x_H_*_.4_	*x_H_*_.5_	*x_H_*_.6_	*x_V_*_.1_	*x_V_*_.2_	*x_V_*_.3_	*x_V_*_.4_	*x_V_*_.5_	*x_V_*_.6_
*B_A_* ^(1,2)^	2.42	1.15	1.16	2.75	0.61	1.62	2.53	0.36	2.56	0.70	0.56	0.86
*B_A_* ^(1,4)^	1.22	2.20	1.14	2.91	0.57	1.62	11.3	2.30	0.73	2.07	1.79	0.84
*B_A_* ^(1,1)^	2.08	0.72	1.33	2.82	0.36	1.27	5.03	4.87	0.82	0.62	1.32	N/A *
*B_A_* ^(2,1)^	1.42	1.43	2.50	1.00	0.44	N/A *	5.12	4.64	0.61	1.26	0.96	N/A *
*B_A_* ^(3,1)^	0.65	7.31	2.22	0.92	N/A *	N/A *	8.02	2.49	0.60	0.58	0.97	N/A *

* Parameter is not present in the structure model.

**Table 4:** Dimensions of the ***B****_A_*^(2,4)^ coupler re-designed for different substrates.

Substrate	*x_H_*_.1_	*x_H_*_.2_	*x_H_*_.3_	*x_H_*_.4_	*x_H_*_.5_	*x_H_*_.6_	*x_V_*_.1_	*x_V_*_.2_	*x_V_*_.3_	*x_V_*_.4_	*x_V_*_.5_	*x_V_*_.6_
Arlon AD250C	1.87	1.87	2.50	1.03	1.00	N/A *	19.57	1.89	1.32	1.91	1.10	1.44
Arlon AD300C	1.18	0.39	2.49	1.95	0.66	N/A *	14.33	1.20	1.01	1.34	1.24	1.76
FR-4	1.72	1.48	2.50	1.00	0.74	N/A *	13.70	1.66	1.39	1.72	1.08	1.03

* Parameter is not present in the structure model.

**Table 5:** Performance characteristics of the re-scaled ***B****_A_*^(2,4)^ designs.

Substrate	*BW* [MHz]	Δ*C* @*ω*_0_ [dB]	∠(*S*_21_/*S*_31_) @*ω*_0_ [°]	Dimensions [mm × mm]	Size [mm^2^]
Arlon AD250C	80	0.12	88.6	15.5 × 58.9	915
Arlon AD300C	72	0.04	88.4	13.5 × 42.2	569
FR-4	70	0.09	88.3	14.0 × 48.9	686

**Table 6:** Comparison of database-inspired design frameworks.

Framework	Size Reduction	Cost	Flexibility	Automation
[16]	–	–	+	–
[32]	+	–	+	+
[72]	+	+	+	+
This work	+	++	++	++

**Table 7:** Comparison of coupler BA(3,1) with benchmark structures.

Structure	*ω*_0_ [GHz]	*ε_r_*	*h* [mm]	BW [%]	Dimensions [mm × mm]	Dimensions [*λ*_g_ × *λ*_g_]	Size [*λ*_g_^2^]	Miniaturization [%] *
[28]	0.5	4.30	1.58	9.6	62.5 × 69.1	0.19 × 0.21	0.0392	35.9
[26]	2.4	4.70	0.80	6.7	11.6 × 13.8	0.17 × 0.21	0.0361	41.0
[30]	2.1	2.54	0.50	3.8	22.2 × 14.9	0.23 × 0.15	0.0341	44.2
[20]	2.4	2.20	0.13	4.7	13.9 × 14.2	0.15 × 0.16	0.0236	61.3
[31]	1.0	10.2	1.27	11.3	14.6 × 20.2	0.13 × 0.18	0.0224	63.3
[73]	1.0	2.94	0.76	8.1	26.6 × 30.9	0.14 × 0.16	0.0217	64.5
[18]	2.4	3.48	0.17	11.0	8.90 × 8.90	0.12 × 0.12	0.0140	77.1
[24]	1.0	3.50	0.51	9.2	17.7 × 18.3	0.10 × 0.10	0.0099	83.8
[29]	0.9	3.38	0.51	8.4	14.7 × 22.7	0.07 × 0.11	0.0080	86.9
This work	1.0	3.38	0.81	9.9	7.70 × 56.4	0.04 × 0.30	0.0132	79.6

* Calculated w.r.t. conventional 3-dB BLC implemented on RO4003C with footprint of 0.0611 *λ_g_*^2^.
